# Valley depolarization in monolayer WSe2

**DOI:** 10.1038/srep15625

**Published:** 2015-10-22

**Authors:** Tengfei Yan, Xiaofen Qiao, Pingheng Tan, Xinhui Zhang

**Affiliations:** 1 State Key Laboratory of Superlattices and Microstructures, Institute of Semiconductors, Chinese Academy of Sciences, P.O. Box 912, Beijing, 100083, People’s Republic of China

## Abstract

We have systematically examined the circular polarization of monolayer WSe2 at different temperature, excitation energy and exciton density. The valley depolarization in WSe2 is experimentally confirmed to be governed by the intervalley electron-hole exchange interaction. More importantly, a non-monotonic dependence of valley circular polarization on the excitation power density has been observed, providing the experimental evidence for the non-monotonic dependence of exciton intervalley scattering rate on the excited exciton density. The physical origination of our experimental observations has been proposed to be in analogy to the D′yakonov-Perel′ mechanism that is operative in conventional GaAs quantum well systems. Our experimental results are fundamentally important for well understanding the valley pseudospin relaxation in atomically thin transition metal dichalcogenides.

Valley pseudospin, which labels the degenerate energy extrema in momentum space, draws increasing attention with the investigation on two-dimensional (2D) materials as a potential controllable degree of freedom in future electronics. The concept of valleytronics, to analogize the spintronics, has been proposed to make use of this degree. Atomically thin transition metal dichalcogenides (TMDCs) is a kind of newly developed 2D materials, with finite direct band gap at the edges of the Brillouin zone at *K*_+_ and *K*_−_ in monolayer flakes^1^,^2^,^3^,^4^. Monolayer TMDCs exhibit a locking between the light circular dichroism and valley pseudospin due to the broken inversion symmetry and large spin-orbit coupling, making them perfect platforms to investigate valleytronics^5^,^6^. Previous works have demonstrated the injection and detection of valley polarization in monolayer TMDCs using photoluminescence (PL) technique^7^,^8^,^9^,^10^,^11^,^12^. Valley polarization relaxation has also been investigated by the time-resolved photoluminescence(TRPL) technique^12^,^13^, transient reflectance spectrum^14^,^15^,^16^ and transient Kerr rotation technique^17^,^18^. The reported exciton valley polarization relaxation is surprisingly fast, which is in the picosecond range. And the relaxation process has been shown to be sensitive to temperature, excitation photon energy and sample treatment method.

The previous studies have excluded the 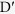
yakonov-Perel′ (DP) and Elliott-Yafet (EY) mechanisms to be responsible for the efficient spin relaxation in monolayer MoS_2_, as the out-of-plane component of exciton spin caused by these mechanisms has been calculated to be in the order of nanoseconds^1^,^19^, which is much longer than the exciton lifetime. Recently, a Maialle-Silva-Sham (MSS) mechanism caused by the intervalley electron-hole (e–h) exchange interaction has been suggested to dominate the spin and valley relaxation in monolayer MoS_2_ and WSe_2_^1^,^17^,^20^. In this work, we report a systematic circular dichroism resolved photoluminescence study on monolayer WSe_2_ at different temperatures with widely tuned laser excitation wavelength and power intensity. Our experimental results confirm the important role of the exciton exchange interaction on the valley polarization relaxation. Though the measured valley polarization has been observed to decrease with increasing excitation intensity at certain excited exciton density range, as previously reported^13^, a surprising non-monotonic dependence of valley circular polarization on the excitation power density is observed by varying the exciton density up to four orders of magnitude in our experiment. The non-monotonic dependence suggests the same scenario here as that of the DP-governed spin relaxation in conventional GaAs quantum well structures. The observed non-monotonic dependence of valley polarization relaxation has been proposed to originate from a close analogue of the DP mechanism, in which the exciton intervalley scattering rate is a non-monotonic function of exciton density with a minimum corresponding to the crossover from the non-degenerate regime to the degenerate one by increasing exciton density.

## Results

Since the degenerated energy level of A exciton in *K*_+_ and *K*_−_ valley corresponds to the opposite spin originating from the broken inversion symmetry, spin flips of electrons and holes are expected to be essential for the intervalley scattering. The intravalley scattering is energetically forbidden due to the large spin split at K point for TMDC monolayers. It is known that the degree of circular polarization is estimated based on the formula^9^:


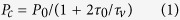


in which *P*_0_ is the initial polarization degree, *τ*_0_ is the exciton lifetime, so the intervalley scattering time is expected to increase monotonically with the intervalley scattering time *τ*_*v*_. Thus the degree of PL circular polarization is associated with the issue of the intervalley scattering time.

**Figure 1:**
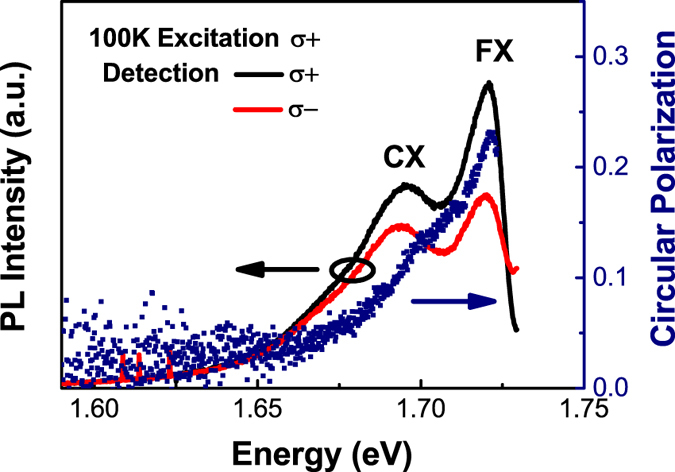
PL response and circular polarization degree of monolayer WSe_2_ excited with pumping energy of 1.77 eV at 100 K. The circular polarization degree is plotted in blue dots.

**Figure 2:**
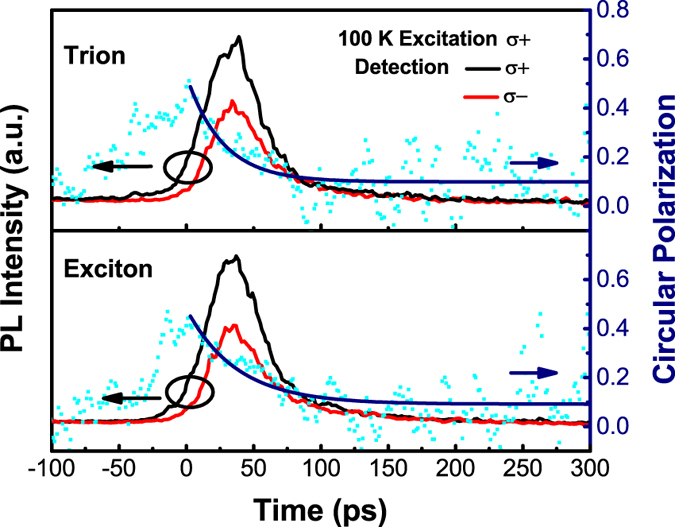
The time-resolved circularly-polarized PL response of both exciton and trion at 100 K with pumping of 1.68 eV. The valley polarization lifetime is obtained by fitting the exponential decay function as plotted with blue solid lines.

At temperatures below 180 K, two individual peaks are easily distinguished in the steady-state PL response of monolayer WSe_2_ as shown in Fig. 1, in which the details of typical PL response excited with the pumping energy of 1.77 eV at 100 K are displayed. The higher energy peak is attributed to the A exciton recombination. While the lower energy PL peak, which has been studied a lot, is related to the trion recombination^12^,^21^,^22^. The time-resolved circularly polarized PL response of both the exciton and trion are measured at 100 K, as shown in Fig. 2, revealing the exciton valley polarization relaxation time to be approximately 38 ± 9 ps and trion valley polarization relaxation time being 27 ± 7 ps, which are obtained by subtracting the nonzero background before fitting with an exponential decay function. The obvious nonzero background of the time-resolved circular polarization may indicate the long-lived residue valley polarization as previously observed and discussed in refs 12,20. The longer valley relaxation time of exciton than trion suggests the higher PL circular polarization of exciton in steady-state measurement, as observed in Fig. 1.

Previous studies have suggested that the valley depolarization is governed by the MSS mechanism^1^,^17^,^20^,^21^,^23^, in which the intervalley e-h exchange interaction results in the intervalley bright exciton transition by virtual recombination of an exciton in *K*_+_ valley and generation in the *K*_−_ valley. As has been theoretically pointed out^20^, the long-range e-h exchange interaction acts as a momentum-dependent effective magnetic field Ω(*p*), with *P* being the center-of-mass momentum of the A excitons. Similar to the DP mechanism, the spins of excitons with different center-of-mass momentums precess around the effective magnetic field with different frequencies, leading to a free-induction decay as that of spin relaxation induced by the inhomogeneous broadening caused by randomly-oriented effective spin-orbit coupling field. The precession frequency due to the momentum-dependent effective magnetic field resulting from the long-range exchange interaction between the two exciton spin states is written as^20^: 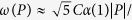
ħ, where C and *α*(1) are material related parameters. Similarly, in analogy to the DP mechanism, the exciton momentum scattering suppresses the inhomogeneous broadening in the strong scattering regime, following the relationship of  20:


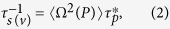


where 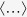 denotes the ensemble average and 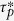 represents the momentum relaxation time of exciton via scattering.

**Figure 3:**
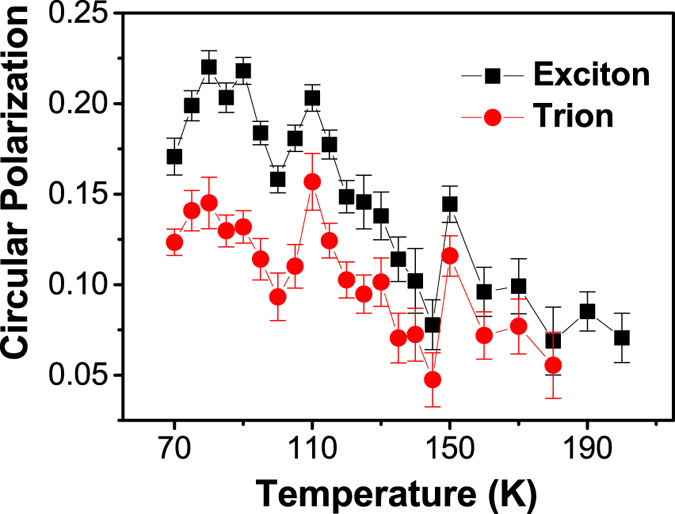
The temperature dependent circular polarization degree of both exciton and trion measured with pumping energy of 1.77 eV. The error bars of the circular polarization degree are given by taking into account both the circular polarization degree accuracy of our measurement system (≥98%) and the deviation of scattered data from the averaged value at each temperature.

The circular polarization of monolayer WSe_2_ as a function of temperature (excited at 1.77 eV) is investigated with the result shown in Fig. 3. As is clearly seen, the circular polarization degree decreases as temperature increases, which is consistent with previous works^8^,^13^,^17^. This can be understood as the momentum-dependent effective magnetic field Ω(*P*) is expected to increase with temperature due to the enhanced exchange interaction, making spin precession and decoherence faster, thus reducing the exciton intervalley scattering time, leading to smaller PL circular polarization.

**Figure 4:**
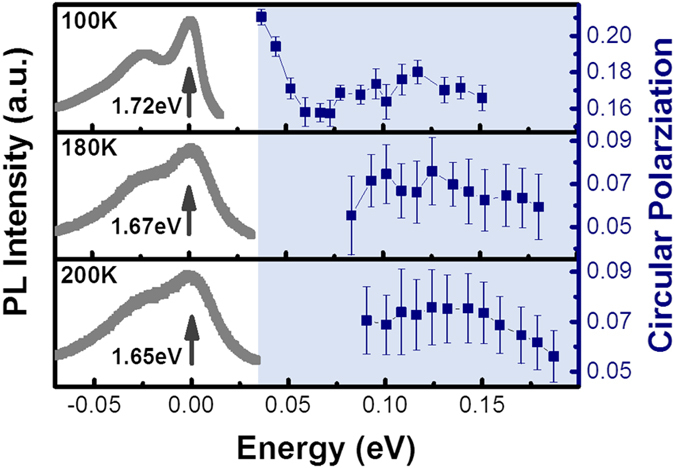
The circular polarization degree as a function of the energy separation between the A exciton and excitation laser, shown in the blue-colored regime on the right. The grey lines on the left are the PL response at different temperatures, and the dark arrow indicates the detection energy. To eliminate the temperature-induced bandgap reduction, the A exciton PL emission peaks at different temperatures (at which energy we detect the circular polarization) are set to be zero. The error bars of the circular polarization degree are given by taking into account both the circular polarization degree accuracy of our measurement system (≥98%) and the deviation of scattered data from the averaged value at each excitation energy.

The excitation laser energy dependent PL circular polarization is investigated at different temperatures as well, with the results summarized in Fig. 4. Here a supercontinuum white light source (Fianium Ltd., model: SC450-2) has been employed together with a Semrock tunable bandpass filter to select the desired excitation laser energy. As can be seen in Fig. 4, when energy separation between A exciton and the excitation laser is smaller than 130 meV, the circular polarization increases with the excitation laser energy. This may be related to the complicated exciton fine structures^24^. While when the energy separation between A exciton and the excitation laser is larger than 130 meV, PL circular polarization is observed to decrease when increasing the energy separation. Similar trend has also been observed in previous works in MoS_2_^10^,^13^ and WSe_2_^24^. This can also be understood based on the intervalley electron-hole exchange interaction model as the center-of-mass momentum of the A exciton obeys^20^:


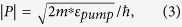


here *ε*_*pump*_ is the excitation laser energy. Thus 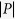 is propotional to the energy separation between A exciton and the excitation laser. The intervalley exciton precession frequency gets larger when *ε*_*pump*_ increases. Hence, the valley(spin) relaxation time decreases with increasing excitation energy, *i.e.*, lower PL circular polarization.

**Figure 5:**
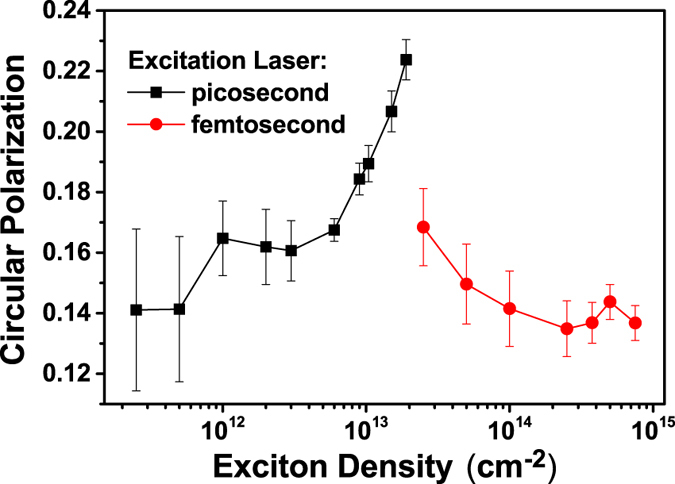
The PL circular polarization degree measured at 100 K as a function of the injected exciton density, with the laser excitation energy of 1.77 eV. The picosecond and femtosecond laser are used to achieve different excited exciton densities, respectively. The error bars of the circular polarization degree are given by taking into account both the circular polarization degree accuracy of our measurement system (≥98%) and the deviation of scattered data from the averaged value at each excitation density.

To explore the influence of exciton density on the exciton valley polarization, the previously mentioned supercontinuum white light with the pulse duration of 40 ps and a femtosecond Ti:Sapphire laser (Coherent Inc., model: Chameleon) with a pulse duration of ~150 fs are used to increase exciton density in a wide order of magnitude. The estimation of the exciton density under excitation of two different laser systems is presented in Fig. S1 in the supplementary material. The circular polarization of monolayer WSe_2_ is first examined with optical pumping at 1.77 eV with picosecond laser excitation by increasing the peak power intensity up to 10^8^ *MW*/*cm*^2^, which corresponds to a photo-generated exciton density of about 10^14^ *cm*^−2^. A typical result measured at 70 K is presented in Fig. S2 in the supplementary material. Surprisingly, a non-monotonic dependence of circular polarization on exciton density is observed: one can see that the PL circular polarization first increases at relatively low exciton density and then falls at high exciton density with a maximum appearing at ~5.0 × 10^13^ *cm*^−2^. However, the monolayer flakes can be occasionally burned under such high average laser power density. In order to have a repeatable and complete examination of exciton density denpendent valley polarization, we thus continued this study by using the femtosecond laser excitation to furthermore increase the exciton density while keeping the average excitation power low enough to avoid damaging the sample. A more complete, non-monotonic dependence of valley polarization on exciton density can be well repeated as shown in Fig. 5, which is carried out at 100 K. Our observation is different from the previous work^13^, in which only the decreased circular polarization with increasing excitation power has been observed, with the excitation laser pulse duration of 1.6 ps and exciton density higher than 10^13^ *cm*^−2^, though a similar trend can be repeated in our experiment (shown in red dots in Fig. 5) with exciton density higher than 10^13^ *cm*^−2^. Meanwhile, TRPL measurements within the tuned excitation intensity range have also been taken in order to investigate the excitation intensity dependent exciton lifetime *τ*_0_ and valley lifetime *τ*_*v*_. It is found that the exciton lifetime *τ*_0_ does not depend on the excitation intensity within the examined excitation power intensity, while the excitation intensity dependent polarization relaxation time is hardly estimated due to the poor signal-to-noise ratio and the limitation of the temporal resolution (20 ps) of the Hamamatsu streak camera system we used. However, based on equation (1), the excitation intensity dependent scattering rate can be deduced to exhibit a non-monotonic dependence on the exciton density.

## Discussion

In the past decade, there have been large number of theoretical and experimental studies for spin dynamics in III-V group semiconductors^25^,^26^,^27^, in which DP mechanism has been revealed to dominate the spin relaxation process. The non-monotonic dependence of spin relaxation time on carrier density in GaAs quantum well structures has been previously observed^28^,^29^,^30^. Since the spin relaxation time is inversely proportional to the momentum scattering time 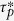, *i.e.*, motional narrowing, the spin relaxation time increases with carrier density in the non-degenerate (low carrier density) regime where 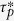 decreases with increasing carrier density, whereas the inhomogeneous broadening 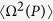 barely changes since carrier distribution function is described with the Boltzmann distribution^20^,^27^,^31^,^32^. However, in the degenerate (high carrier density) regime, the inhomogeneous broadening is greatly enhanced with electron density, leading to the decreased spin relaxation time with carrier density.

For the case of monolayer WSe_2_ investigated in this work, in the low excitation regime, the laser excited exciton density is low, *i.e.*, the non-degenerate regime. Considering that the excited excitons within the non-degenerate regime obey the Boltzmann distribution, which is the same as that of electrons, we propose a similar relaxation process of valley pseudospin on exciton density as that of electron spin in GaAs quantum wells. The inhomogeneous broadening 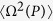 originating from the long-range exchange interaction between electron and hole has weak dependence on the exciton density in non-degenerate regime, whereas the center-of-mass momentum scattering rate 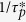 increases with increasing exciton density, hence the spin (valley) relaxation time *τ*_*v*_ increases with excitation density because of the motional narrowing, which is exactly what we observed experimentally as shown in Fig. 5. At higher excitation density, the large number of excited excitons relax to the higher bands of the energy band as lower bands are filled. These higher-energy excitons, namely hot excitons, possessing larger center-of-mass momentum, precess with larger frequency due to the long-range e-h exchange interaction, leading to an enhanced inhomogeneous broadening 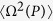 and decreased valley(spin) relaxation time *τ*_*v*_. As a result, a peak for excitation density dependent valley polarization would appear at the crossover between the non-degenerate and degenerate exciton distribution regimes, as we observed in Fig. 5.

In summary, the circularly polarized PL response of monolayer WSe_2_ has been systematically examined at different temperatures, excitation energies and intensities. The experimentally determined valley polarization and its relaxation provide evidence for the depolarization mechanism governed by the intervalley electron-hole exchange interaction. More importantly, we observe a non-monotonic dependence of valley polarization on the excitation power density, providing the experimental evidence for the non-monotonic dependence of exciton intervalley scattering rate on the excitation power density. The relaxation of valley pseudospin in monolayer TMDCs is thus proposed to exhibit the similar scenario as that of the DP-governed spin relaxation in conventional GaAs quantum well structures.

## Methods

The WSe_2_ flakes are fabricated by mechanical exfoliation with adhesive tape from a bulk crystal (2D semiconductors Inc.) onto *SiO*_2_/*Si* substrates. Monolayer WSe_2_ flakes are identified by optical contrast under a microscope first and then confirmed via Raman and PL measurements as presented in our previous work^22^. The circular polarization resolved PL measurements is carried out by a microscopic confocal PL setup^8^. PL response, collimated by a 50× objective lens, passes through a quarter wave plate and a beam-displacer to be circularly separated out. Excited by a *σ*^+^ circularly polarized laser beam, the degree of PL circular polarization is determined by 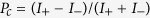, where *I*_+_ and *I*_−_ correspond to the PL intensity of *σ*^+^ and *σ*^−^ components, respectively. A supercontinuum white light source (Fianium Ltd., model: SC450-2) and femtosecond ultrashort pulsed laser (Coherent Inc., model: Chameleon) are used respectively for the purpose to vary the excitation peak density in a wide range. For the temperature dependent measurement, the sample is mounted in an Oxford nitrogen-flow microscope cryostat (Microstat HiRes II).

## Additional Information

**How to cite this article**: Yan, T. *et al.* Valley depolarization in monolayer WSe_2_. *Sci. Rep.* **5**, 15625; doi: 10.1038/srep15625 (2015).

## Supplementary Material

Supplementary Information
