# Supplementary material for: Valley depolarization in monolayer WSe2
Source: Sci Rep. 2015 Oct 22;5:15625. doi: 10.1038/srep15625 (PMC4614989; doi:10.1038/srep15625)
Supplement: Supplementary Information [file srep15625-s1.pdf]

Valley depolarization in monolayer WSe_2

Tengfei Yan, Xiaofen Qiao, Pingheng Tan & Xinhui Zhang

S1. Exciton density estimation excited by different laser systems

To estimate the exciton density excited by different lasers, a rate equation was used:

$$\frac{dN}{dt} = -\frac{N}{\tau} + Ae^{-(t-t0)^2/2\sigma^2}$$

Where N is the exciton density and τ is the exciton lifetime. The second term in the above equation denotes the excited exciton increase described by a Gaussian function with parameters of the excitation laser, where σ is related to the laser pulse width and A describes the density of exciton excited by a laser pulse which is associated with the absorption coefficient of WSe_2, average pulse energy and pumping photon energy. The exciton density is thus estimated under excitation of picosecond (with ~40 ps pulse width) and femtosecond (with ~150 fs pulse width) lasers, respectively, as presented in Fig S1, in which laser excited exciton density is plotted as a function of time with the same average laser power of 1 µW for both laser systems.

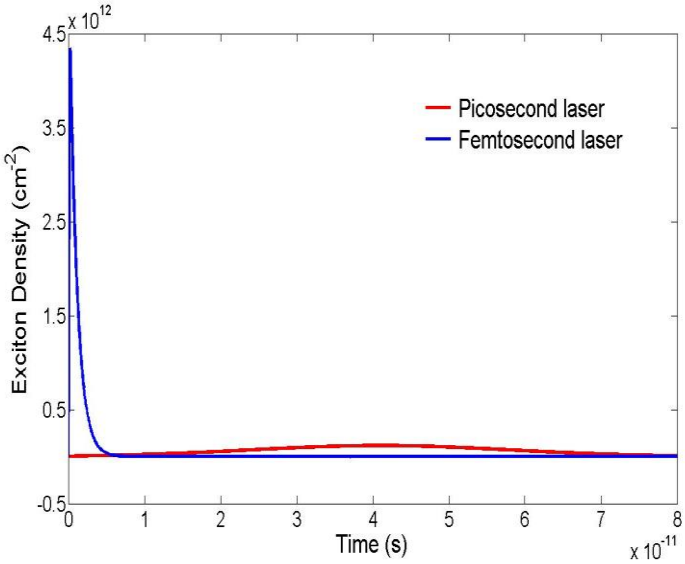

Fig. S1. Calculated exciton density as a function of time under excitation by different laser systems. The blue and red lines correspond to the femtosecond and picosecond laser excitation, respectively.

S2. Exciton density dependent photoluminescence circular polarization

The circular polarization of monolayer WSe_2 was examined with optical pumping at 1.77 eV with picosecond laser excitation first. A typical result of the exciton density dependent circular polarization degree by increasing the excitation power density of the picosecond laser, as described in the main text, is presented in Fig. S2. A clear peak of the circular polarization degree is seen at ~5×10^{13} cm^{-2} with increasing exciton density measured at 70 K. Note that the different circular polarization degree presented in Fig. 5 and Fig. S2 is due to the different flakes we used in the experiment. As mentioned in the main text, monolayer flakes tend to be damaged at high excitation laser intensity, thus the absolute circular polarization degrees slightly varies from piece to piece when examining different flakes.

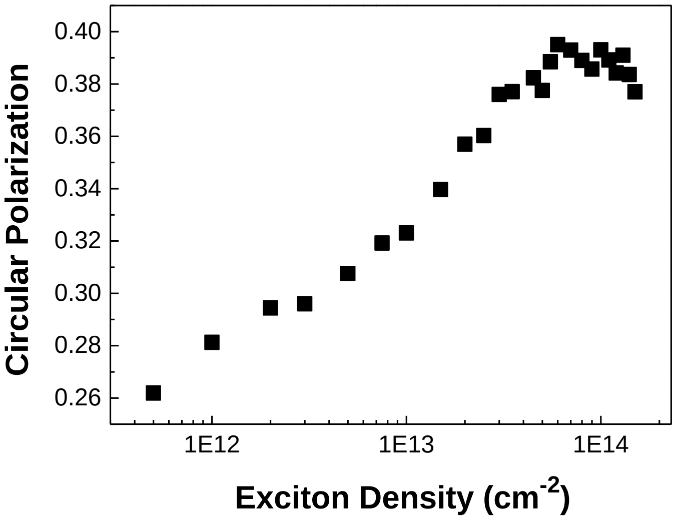

Fig. S2. The PL circular polarization degree as a function of the injected exciton density, excited by the picosecond laser only at 70 K and pumping energy of 1.77 eV.
